# Immune response to hepatitis B vaccine in patients who lost hepatitis B surface antigen during follow up

**Published:** 2011-06-01

**Authors:** Georgios Zacharakis, Nikos Viazis, Dimitrios G Karamanolis

**Affiliations:** 1 Second Department of Gastroenetrology, Evangelismos Hospital, Athens, Greece

**Keywords:** Hepatitis B surface antigen, Hepatitis B vaccine

Dear Editor,

We read with interest the article by Taheri et al. regarding the efficacy of Hepatitis B vaccine in those who lost Hepatitis B surface antigen (HbsAg) during follow up [1]. As the authors mentioned, a protective anti-HBs level developed in 24% of chronic HBsAg-positive subjects who had already lost their HBsAg after hepatitis B vaccination, and the remaining cases need to be monitored for occult HBV infection. Subjects with no response after hepatitis B vaccination may have low levels of HBsAg or have immunologic tolerance to hepatitis B vaccination and no ability to produce anti-HBs antibody as reported previously [2]. Detection of HBV DNA in the absence of a detectable HBsAg level and occasionally other HBV serologic markers is termed occult hepatitis B (OHB) [3]. These patients can not only transmit HBV to others but also may progress to chronic hepatitis, cirrhosis, and hepatocellular carcinoma. The prevalence and outcomes of OHB in chronic HBV-infected individuals have not yet been reported. On the other hand, patients who lost HBsAg and have not seroconverted to anti-HBs with no detectable HBV DNA are frequently seen in clinical practice, and the outcomes for this group of patients are not clear. A study by our research group on the long-term outcomes of chronic hepatitis B surface antigen (HBsAg) carriers in the general population in northeastern Greece showed that HBsAg to anti-HBs seroconversion was observed in 10 out of 195 (5.1%) patients at the inactive carrier state, with an estimated annual prevalence rate of 1%. Additionally, six patients lost HBsAg (3.1%) without developing anti-HBs immunity. All patients who lost HBsAg during the follow up period were HBeAg negative and anti-HBe positive and had undetectable serum HBV-DNA and normal ALT levels [4]. In another study, we determined that the frequency chronic HBV patients with isolated anti-HBc was 6% in the general population of northeastern Greece, where HBsAg endemicity is about 3% [5]. Serum HBV-DNA levels were less than 2,000 IU/ml and were detected in 9 out of 93 (9.7%) anti-HBc positive, anti-HBe positive individuals, of whom 3 developed anti-HBs during the follow up period despite the persistence of serum HBV-DNA [6]. In patients with detectable levels of HBV-DNA, no mutation was detected in the S gene. These subjects either have chronic HBV infection but lost HBsAg over time or resolved HBV infection with a decrease in anti-HBs antibody levels below 10 IU/L [6]. For patients with isolated anti-HBc antibody who receive the hepatitis B vaccination, several studies have reported significant anti-HBs levels of 91%-96% of the subjects [2][7][8][9]. Lok et al. reported no response rate after three doses of hepatitis B vaccine in 28% of 32 subjects with isolated anti-HBc antibody [2]. Lai et al. reported no anti-HBs response in 22.9% of 48 cases with isolated anti-HBc after three doses of the hepatitis B vaccination [9]. Our data are in agreement with the results of the above studies and suggest a relatively low percentage (26%) of no anti-HBs response in patients who are anti-HBc positive only (unpublished data). Although Taheri et al. found that 24% of chronic HBsAg-positive subjects who lost HBsAg developed anti-HBs after receiving the hepatitis B vaccination, we have to take into account spontaneous HBsAg seroconversion. Indeed, according to our results, 47.8% (163/341) of individuals with detectable anti-HBc levels at presentation developed anti-HBs immunity (annual rate of 9.5%) and had undetectable serum HBV-DNA during the observation period of up to 17 years [10]. Our data are in agreement with the results of Taheri et al.[1] that chronic HBsAg-positive cases who lost their HBsAg and are negative for HBV DNA mostly responded to hepatitis B vaccination. Additionally, these patients differed from the remaining patients who lost HBsAg during follow up and were positive for HBV DNA, who still might have OHB and must be followed up with.
